# Toward Interdisciplinary Synergies in Molecular Communications: Perspectives from Synthetic Biology, Nanotechnology, Communications Engineering and Philosophy of Science

**DOI:** 10.3390/life13010208

**Published:** 2023-01-11

**Authors:** Malcolm Egan, Murat Kuscu, Michael Taynnan Barros, Michael Booth, Antoni Llopis-Lorente, Maurizio Magarini, Daniel P. Martins, Maximilian Schäfer, Pasquale Stano

**Affiliations:** 1 Univ Lyon, INSA Lyon, INRIA, CITI, 69621 Villeurbanne, France; 2 Department of Electrical and Electronics Engineering, Koç University, Istanbul 34450, Turkey; 3 School of Computer Science and Electronic Engineering, University of Essex, Colchester CO4 3SQ, UK; 4 Department of Chemistry, University College London (UCL), London WC1H 0AJ, UK; 5 Instituto Interuniversitario de Investigación de Reconocimiento Molecular y Desarrollo Tecnológico (IDM), CIBER de Bioingeniería, Biomateriales y Nanomedicina (CIBER-BBN), Universitat Politècnica de València, Camino de Vera, 46022 València, Spain; 6 Department of Electronics, Information and Bioengineering (DEIB), Politecnico di Milano, 20133 Milan, Italy; 7 Walton Institute for Information and Communication Systems Science, South East Technological University (SETU), X91 P20H Waterford, Ireland; 8 Institute for Digital Communications, Friedrich-Alexander-Universität Erlangen-Nürnberg (FAU), 91058 Erlangen, Germany; 9 Department of Biological and Environmental Sciences and Technologies (DiSTeBA), University of Salento, 73100 Lecce, Italy

**Keywords:** molecular communication, biological communication, information

## Abstract

Within many chemical and biological systems, both synthetic and natural, communication via chemical messengers is widely viewed as a key feature. Often known as *molecular communication,* such communication has been a concern in the fields of synthetic biologists, nanotechnologists, communications engineers, and philosophers of science. However, interactions between these fields are currently limited. Nevertheless, the fact that the same basic phenomenon is studied by all of these fields raises the question of whether there are unexploited interdisciplinary synergies. In this paper, we summarize the perspectives of each field on molecular communications, highlight potential synergies, discuss ongoing challenges to exploit these synergies, and present future perspectives for interdisciplinary efforts in this area.

## 1. Introduction

Molecular communications is an emerging area of communication theory that involves using molecules, whether synthetic or natural, to encode information [1]. Originally developed by communication engineers for technological goals, such as supporting in-body communications, a vision has now arisen as a motivation for the design of interactions between both synthetic and biological components. A key feature of this vision is its emphasis on biological or chemical systems that perform a communication function. This is achieved by decomposing chemical or biological systems into components, known as transmitters, channels, and receivers, wherein the transmitter seeks to induce specific changes in the receiver via the propagation of chemical messengers. The study of systems from such a communication perspective is then sustained by techniques from information and communication theories.

In parallel, synthetic biology and certain areas of applied chemistry and nanotechnology have established new means of supporting interactions within and between synthetic and biological systems [2,3]. In general, work in these areas is performed from a mechanistic perspective to understand how different components of a system function and how they can be improved. Experiments often focus on the mere achievement of the planned interaction. On the other hand, recently new motivations have arisen from wider ranging visions for the potential of molecular communications both at the practical and theoretical levels. For example, a quite evocative scenario considers a wetware chemical version of the famous Turing test, the cellular imitation game [4], as a tool for monitoring the progress in building artificial systems (e.g., synthetic cells or similar nanoparticles) capable of communicating with biological systems. This suggests the possibility of useful partnerships between communication engineering and synthetic biology or nanotechnology, with very interesting and still unexplored avenues of research, which may also interact with the philosophy of science.

The potential synergies between communication engineering, synthetic biology, nanotechnology, chemistry, and philosophy of science on research involving molecular communications have been rarely discussed and presented explicitly to the wider scientific community. Here, based on a recent workshop dedicated to these themes (see the Acknowledgements), we provide an initial report aiming to fill this gap, therefore promoting the convergence of different disciplines to common themes, questions, and solutions. The novelty of the present discussion mainly refers to the authentic advancements and to the synergies that can arise when specialists from different fields start to think and work together. More specifically, we envision the possible scenarios arising from the introduction of recently developed molecular communication theories and related frameworks (e.g., [5]) on experimental approaches such as those usually carried out in synthetic biology and nanotechnology. Key questions that motivate our interest in these scenarios range from how the mathematical and conceptual tools usually conceived in communication engineering and philosophy of science can aid novel experimental investigations in synthetic biology and nanotechnologies, and, vice versa, how synthetic biology and nanotechnology can play a role—as tailor-made experimental platforms—in the development of innovative technological strategies based on the exchange of chemical signals.

Building on the discussions at the workshop, this perspective article seeks to provide a structured overview of the various perspectives on molecular communications as well as the potential synergies between them. The article also addresses the challenges and obstacles that may impede such interactions and proposes strategies to overcome them. We firmly believe that the cross-fertilization of experimental approaches in synthetic biology and nanotechnology, along with the mostly theoretically informed “molecular communication” view of communications engineers, would unlock new insights and opportunities in molecular communications. This interdisciplinary approach is essential for furthering our understanding of this fundamental and unconventional form of communications and actualizing its full potential.

## 2. Four Perspectives on Biological Communications

### 2.1. Synthetic Biology

Synthetic biology can be defined as the design and the construction of new biological parts, devices, systems, and the re-design of existing, natural biological systems for useful purposes [6,7]. This new research area lies at the intersection of biology and engineering and, since its early days, has been mainly focused on achieving goals for applied research. It does not exclude, however, the interest toward basic science, as the resulting systems can be designed in order to investigate fundamental biological questions, too. In synthetic biology, existing organisms (generally, but not exclusively, microorganisms) are engineered in various ways and for various goals [8,9,10]. Cell-free systems, such as those devoted to in vitro protein synthesis, are also considered valuable tools in synthetic biology [11,12]. Finally, the construction of cell-like systems from scratch represents still another research branch [13,14,15,16]. By the latter approach, which will be the only one discussed in this article, “synthetic (or artificial) cells” can be generated. This is made possible by the co-encapsulation of a specific set of molecules (e.g., DNA, ribosomes, enzymes, metabolites) in artificial microcompartments (e.g., liposomes, polymersomes, droplets, coacervates). Very often, synthetic cells are based on multi-enzyme pathways or on coupled transcription–translation reactions. Despite the name, the resulting structures are actually very rudimentary cell models, and they are not comparable with any biological organism. Current synthetic cells are not alive, but the latest results show that they can display an increasing number of cell-like functions, including the capacity of sending and receiving chemical messages [17,18,19,20]. It is not surprising that a large number of synthetic biology investigations, of any type, rely on molecular communication, making use of appositely designed molecular circuits. Indeed, the crucial importance of communicating—for any biological system—cannot be underestimated, and such a fundamental mechanism has become a target process to be exploited when new applications are devised. If synthetic cells are designed and built to function as smart drug delivery agents (a scenario not yet achieved but described in perspective papers [21,22,23]), it is required that synthetic cells engage biological cells in a communication dynamics. Such a strategy ensures that the behavior of synthetic cells will depend on the environment, which will consist of biological cells and biological molecules of the human body. This environment will provide the chemical signals that are necessary for the synthetic cell to make decisions: for example, to produce a drug *in situ*. Essentially, this process corresponds to perception and action, and it can be realized by synthetic cells via a proper design of their internal chemical network.

### 2.2. Nanomaterials Science and Nanotechnology

Over the last few decades, Nanomaterials Science and Technology have revealed the unique features of simple and composite matter at the nanoscale. A very wide range of artificial nanoparticles have been synthesized by original methods and studied with respect to physical, mechanical, and chemical properties as well as with respect to their interaction with biological systems—from cells in culture to whole organisms. They exhibit very specific interaction patterns that can be readily exploited in industrial, environmental, and medical applications. In the context of molecular communications, nanoparticles exist and exert their function at the same nanoscale where fundamental physicochemical processes occurs. Some of the most appealing properties of nanoparticles in general, apart from their size, include their high specific surface area and the possibility to functionalize them with multiple functional groups such as chemical moieties, DNA strands, enzymes, and antibodies [24,25]. They are, therefore, privileged systems for generating, interfering, altering, amplifying, destroying, etc., any sort of chemical or physical signaling. For these reasons, nanoparticles with advanced capabilities are often referred to as nanomachines (possibly bio-nanomachines) for carrying out specific tasks.

When endowed with communicating capacity, the potential applications of nanomachines have been described elsewhere [5]. For the sake of the present discussion, it is enough to recall that properly designed nanosystems can facilitate a targeted and controlled drug delivery, for instance by improving targeting performances, achieving sustained release, amplifying signals, and performing more complex operations, for example in a cooperative manner [5]. Nanoparticles can play a role for intracellular drug delivery, too. Additional scenarios, all based on molecular communication, can be envisaged, such as lab-on-a-chip technologies, unconventional computing, manufacturing, and environmental applications.

In recent years, several nanotechnology concepts with high potential for molecular communications have emerged. For instance, nanoparticles can be loaded with selected chemicals in their interior, which upon release in response to specific stimuli act as chemical messengers in communication protocols. In this direction, Martínez-Máñez, Llopis-Lorente and colleagues have leveraged the use of porous particles functionalized with molecular gates to design several communication pathways between different nanoparticles. These examples include cascade-like linear communication [26], interactive (feedback) communication and a circular communication network between multiple nanoparticles [3]. The key concept here is that nanoparticles exchange chemical messengers between them in a pre-progammed sequential manner, leading to a collective output response from the final nanoparticle (e.g., release of a reporter or drug) in the network. In addition, nanoparticles have also been engineered to communicate by means of molecular messengers with living microorganisms [27] and to act as nanostranslators to enable communication between different microorganisms [28].

In a different complementary direction, Tuccito and colleagues have demonstrated the use of nanoparticles as chemical messengers for telecommunications [29]. In this approach, nanoparticles travel from the sender point to the receiver, acting as carriers/transmitters of the information, leveraging intrinsic or engineered nanoparticle properties such as fluorescence or magnetism.

Another interesting concept in the area of of nanotechnology is the development of nanomotors or also known as nanobots [30]. Nanomotors are active particles with the ability to exhibit autonomous motion by converting a chemical fuel or external irradiation into self-propulsion. Nanomotors hold great potential in different areas, including nanomedicine [31,32]. One major challenge in this area is to control the collective actuation and swarming behavior of nanomotors. Thus, potential synergies between molecular communications and nanomotors may emerge in the near future to achieve collective control. In addition, nanomotors hold potential to be used in communication protocols as carriers of chemical information.

### 2.3. Communication Engineering

The communication engineering perspective on molecular communications is concerned with applying ideas from information theory and communication networks to biological and chemical systems [1]. In particular, there is a strong emphasis in developing models of biological and chemical systems as well as using tools from information theory and networking to analyze their capability to exchange information between spatially distributed components.

Work in molecular communications is often oriented toward an engineering perspective, which is often described in terms of the vision of the Internet of Bio-Nano Things (IoBNT) [33,34], which aims to develop platforms to connect biological and nanoscale systems in a fashion analogous to the Internet. At the functional level, this perspective separates biological and nanoscale entities (bio-nano things) into three main categories: transmitters, channels, and receivers. A transmitter is the entity that converts information into molecules or molecular structures/patterns. The channel is the media where the molecules propagate through various means. The receiver is the entity that reacts to the molecular information when such is interpreted by it.

The study and design of biochemical systems using the transmitter–channel–receiver model typically adopts the approach initially due to Claude Shannon, who developed the earliest mathematical frameworks for the quantitative analysis of communication and information systems [35]. As such, besides determining whether interactions occur between components, the focus is often on quantitative metrics drawn from information theory. These metrics are used to define fundamental limits for the transmission of messages, and to date, extensive work has been conducted on codes and methods for approaching these limits. For example, channel coding techniques such as Hamming [36] and Reed–Solomon codes [37], which are mostly adapted from conventional electromagnetic communications, have been applied to molecular communications to improve the error rate performance. Some of these techniques have addressed specific challenges of resource-limited bio-nano things communicating with molecules in a fluidic channel, such as addressing the energy consumption problem while improving communication performance [38], or targeting intersymbol interference (ISI) resulting from the memory of the diffusion-based molecular communication channels [39]. The design of an encoder for the Single Parity-Check code and its decoding using genetic circuits techniques is considered in [40], where biochemical simulations were carried out to demonstrate the closeness of error rate performance to that achieved with an electric implementation.

By establishing a mathematical link between the biochemical description of the system and communication metrics at various networking levels, the engineering approach naturally has the potential to inform the design of biochemical systems optimized from communication and information perspectives. Application of this transmitter–channel–receiver model, or the *molecular communication model*, to biochemical systems is finding further practical relevance in life sciences by providing new communication and network biomarkers for the early diagnosis of diseases whose origins can be traced down to biological/molecular communication problems.

Instead of focusing solely on message transmission, the study of *semantic communication* introduces a new paradigm shift toward the definition of a Post-Shannon metric of information [41]. This implies a change from the conventional design to a new one that goes beyond the transmission of purely message bits and considers the meaning associated with the conveyed message. This change of paradigm has the potential to increase communication efficiency and also to open the way for the applicability of new quantitative metrics of information more suitable to describe the interaction between biological systems.

Indeed, Shannon’s primary interest was to define a measure to quantify how much information is generated by a source and reliably transmitted across a communication while specifically avoiding taking the semantic component of information into account. In the semantic communication process, the goal is to accurately recover the meaning of information at the destination. This introduces additional aspects in terms of coding intents and coding methods. In typical application contexts, semantic encoding and decoding can increase the efficiency of communication by minimizing a semantic error measure based on the dissimilarity of meanings. This leads to the definition of the optimal transmission policies to best preserve the meanings of recovered messages, which typically considers the presence of an external entity, i.e., an agent, that can influence the receiver by providing contextual information. In this situation, efficient intent-oriented interactions between communication objects are made possible by the aggregation and extraction of valuable information according to criteria based on semantic measures of information.

### 2.4. Philosophy of Science

Speaking about communication in artificial systems such as synthetic cells or nanoparticles, and in particular when such a process involves living biological entities, too, immediately elicits relevant philosophical questions. In particular, the artificial systems can serve as a platform for investigating which sort of chemical *organization* can generate and support operations and behaviors such as exchanging signals with biological systems. To date, the artificial systems that have been studied from a philosophical viewpoint to understand communication between artificial and biological entities include hardware and software systems (i.e., robotics, AI), but investigations explicitly devoted to wetware chemical systems are missing. Because current developments indicate that synthetic cells or nanoparticles of various types entered the arena of communication via chemical exchanges with biological systems, unprecedented questions quickly arise. Epistemologists are therefore called to identify the new relevant issues that are specific to the chemical communication, chemical information, and chemical organization. For example, the following tasks seem to be relevant: (i) define, analyze, discuss the very concept of communication in wetware artificial systems, in particular by identifying distinction between mere interactions and communication; (ii) define the theoretical frameworks within which these new wetware systems should be theoretically handled; and (iii) understand whether, and in which respect, the specific chemical nature of wetware systems constitute a novel class of dynamical systems, alluding to their capacity of establishing communicative, cognitive, and perceptive features.

## 3. Potential Synergies

A key challenge for interdisciplinary collaboration between the different perspectives on molecular communications is that each perspective entertains a distinct vision for the understanding and application of molecular communication. For example, while synthetic biology focuses on the construction of artificial systems capable of communicating via chemical exchanges with other artificial or with biological systems, the communication engineering approach focuses on the understanding of how cells communicate with each other and exploit the underlying principles in order to use molecular communications for useful purposes. The interests of the latter perspective involve the modulation, detection, and coding techniques implemented by cells, and resource allocation strategies to maintain the reliability of communication in the presence of noise and interference. No perspective is subservient to any other, and all contribute to a deeper understanding and application of chemical communications. It is therefore necessary and fruitful to identify synergies between these perspectives to make progress toward each of the visions.

In particular, we can refer to visions related to technology and visions related to fundamental science, as discussed below. In this section, we identify synergies between the perspectives that may aid progress toward both visions.

### 3.1. Synergies for Progress toward Technological Visions

Molecular communications is a promising candidate for a common language among bio-nano things as it is already employed by natural systems, i.e., exchange of molecules to encode and transfer information. Therefore, this fundamental and long-evolved communication modality has been of great interest to communication engineers, who attempted to reveal its foundations and limitations from the technological perspective [1]. Based on this common language, an ambitious technological vision emerged from the molecular communications community: IoBNT. This engineering framework envisions seamless communication interfaces among bio-nano things, which is an umbrella term covering natural and synthetic biological systems and components as well as artificial nanoscale devices [33]. Communication between heterogeneous components within the IoBNT framework is expected to enable novel healthcare and environmental applications [34].

Communication and networking requirements of the IoBNT applications can inform the design of synthetic cells of practical use, such as signal conversion and Internet connectivity [33,34]. These requirements can be even beyond the capabilities of natural biological cells (especially in the so-called top–down approaches—those based on the modification of living cells) and bring up new challenges for synthetic biology research regarding the seamless integration of several new functionalities to the synthetic platforms. For example, the integration of biology and electronics (based on molecular communications) can enable the exchange of biochemical information through the Internet, which can be applied for the remote sensing and monitoring of biological systems [42]. In addition to that, IoBNT applications will require the conversion of electrical signals into biochemical signals to interface electronic systems with a wide variety of cells in a biological environment. Initial works on this topic have shown the feasibility of miniaturize signal converters for such applications [43,44], but their full integration with natural cells and systems still remains as an open challenge.

At the same time, molecular communication has been naturally adopted in all kinds of synthetic biology approaches, and a number of experiments focused on this subject have been developed. Among them, synthetic cells that communicate with each other or with biological cells show the progress in designing and constructing from scratch cell-like artificial systems with specific (programmable) behavior [17,45,46,47]. These perspectives indicate a potentially strong synergy between communication engineers and synthetic biologists toward enabling the envisioned IoBNT applications. In particular, synthetic biology tools and platforms can be harnessed to validate and refine the molecular communication theories and techniques, which typically lack physical correspondence at present [48]. Analogous synergies between the technological vision of the IoBNT and the science of nanomaterials naturally arise through the use of communicating nanoparticles [3,49].

### 3.2. Synergies for Progress in Fundamental Science

It is typical in fundamental chemical and biological sciences to find mechanistic explanations for the behavior of chemical or biological systems. These kinds of explanations can be achieved either by the direct observation of system components or by attempts to reconstruct closely related systems. Explanation by construction [50] is at the heart of synthetic biology, as it happens—for example—in the case of protocellular models [51,52]. The synthetic approaches can address fundamental questions about biological mechanisms (e.g., identifying the minimal complexity needed to generate a defined behavior), about the overall organization of living systems (e.g., by providing an experimental platform to investigate theories such as autopoiesis and chemoton [53,54,55]), and about the principles of chemical information processing.

Another question particularly prominent in synthetic and physical chemistry is to understand the emergent capabilities of collections of different chemical entities. This question is a key motivation for the design of new nanoparticles with communication capabilites in addition to other motivations drawn from the development of biomedical technology and new materials for various industries.

How can the molecular communications community aid the fundamental sciences to either explain or understand the capability of chemical and biological systems? A tentative step toward answering this question lies in the fact that molecular communications adopts a *functional* perspective of chemical or biological systems. In particular, a system is not viewed only as a number of interacting components but also as a means of reproducing a message (in a general sense) sent from one component to another spatially separated component. In a certain sense, molecular communications considers what a system does as a whole, putting aside mechanistic details. This corresponds to a high-level description of system behavior and thus refers to its functional features.

While molecular communications advocates a systematic application of the functional perspective, this perspective is already present to some extent both in synthetic biology and nanotechnology, but often, it is only discussed and interpreted at a qualitative level just by looking at whether or not a sort of communication took place.

On the other hand, in nanomaterials science, a recent focus has been on nanoparticles that can release chemical messengers able to stimulate other nanoparticles. The capability of these nanoparticles is measured from the functional perspective: that is, whether or not the released chemical messengers are capable of stimulating other nanoparticles.

A key synergy between molecular communications and synthetic biology or nanotechnology is therefore to advocate the systematic application of the functional perspective inherent to molecular communications. The functional communication perspective can indeed drive the design and the construction of new chemical or synthetic biology systems. These new systems can overcome current approaches within synthetic biology and nanotechnology by satisfying requirements based on more refined quantitative metrics of performance than simply whether a given interaction occurs or not. Moreover, the typical procedural approach employed in communication theory, i.e., the probabilistic one, is rarely applied in current experimental approaches. Therefore, setting the stage for truly interdisciplinary studies of chemical communications will further contribute to deploy more sophisticated conceptual and modelling tools in chemistry and biology studies.

The functional communication perspective also provides a basis for the comparison of synthetic biological or chemical systems that are—by definition—very different in their components. For example, nanoparticles with different chemical composition are typically studied by different laboratories. By adopting the functional communication perspective, a single laboratory or groups of collaborating laboratories have new motivations to study multiple nanoparticle systems and compare their capabilities within the context of their performance measured by molecular communication metrics. Such an approach can facilitate the dissemination of specialized expertise on nanoparticles and suggest new structures not evident when only expertise related to a small family of nanoparticles is available. In addition, it will endow the whole scientific community with a sort of conceptual tool for monitoring the progress and advancements in the field.

A third synergy that can arise from interactions between molecular communications and synthetic biology or nanotechnology is a new framework to study transient behavior in complex systems. For example, in nanoparticle systems with sending and receiving nanoparticles, understanding the release of a cargo over time both by the senders and the receivers is of key importance [3]. This kind of problem has been widely considered in molecular communications in the form of coding schemes. A natural question is therefore whether adopting tools and modeling approaches from molecular communications can provide a formal framework to develop new requirements for synthetic biological and chemical systems to guide experimental design.

A final remark refers to the role that molecular communication approaches, based on the solid information and communication theories, can play in the emerging field of semantic information (in this case applied to chemical and biological systems). For example, a recent study has highlighted a possible strategy that makes use of mutual information, which is a Shannon information metric of the mutual dependence between two random variables, in a communication scenario made of physical agents situated in an environment [56]. The approach resonates well with the interests, the tools, and the goals of molecular communication and synthetic biology [41].

## 4. Challenges in Connecting Communities

Even if there are numerous synergies between the problems and techniques that are the focus of each community, it is also clear that there are important challenges that must be addressed before these synergies can be realized. Two major challenges can be identified: a lack of a common language and gaps between the mathematical/conceptual frameworks and experimental work.

### 4.1. Lost in Translation

Depending on whether one works in synthetic biology or chemistry, communication engineering, or in the philosophy of science, the terms “communication” and “information” often have very different meanings. For example, what does a chemical communication system consist of? For a nanomaterials scientist, a communication system may consist of a nanoparticle that responds to an stimulus and subsequently releases a cargo that acts as a chemical messenger for another nanoparticle or living cell. On the other hand, for philosophers of science, the interest in communication may consist in the conceptualization of the type of organization that emerges when a sender produces a signal and a receiver perceives it or even responds to it [57]. In which terms such a signal makes sense to the sender and to the receiver, and at what extent “meaning” can be genuinely generated in artificial systems are typical questions to deal with, in collaboration with scholars from different areas, only after a common vocabulary has been developed.

In terms of information, there are now many notions, both “statistical” (e.g., Shannon metrics) and “semantic”. While statistical information is reasonably well defined, this is not the case for semantic information. For example, in the philosophy of science, semantic information views signals as representations. On the other hand, recent work in communications engineering often views semantic information as generalizations of statistical information. For physicists, a recent approach sees semantic information as the part of statistical (syntactic) information that is causally necessary for the system to maintain its own existence [56]. Even though the definition of semantics in molecular communication can be further developed, there is the potential to explore the limits of digital communications when applied to molecular communication with analog nature. A more refined application of communication theory models to biological systems, here synthetic, may lead to novel communications methods.

If such basic questions of terminology are not resolved, it is clear that any interaction between the communities will be limited. Indeed, without clearer definitions, it is also not obvious that the communities are even working on related problems. As both communities continue to evolve over the years, it is important that these issues are closely monitored to ensure that the relationships between both communities are maintained at the highest levels. In this respect, we hope for more frequent occasions of contact, debate, and reciprocal knowledge. The virtual workshop we are commenting here can be seen as a first step in this direction.

### 4.2. The Gap between Mathematical/Conceptual Frameworks and Experimentation

A typical synthetic biologist is concerned with the question: does a synthetic cell produce a certain behavior? This question concerns whether or not a synthetic cell produces a particular response (e.g., chemical or fluorescent) in another synthetic or natural cell upon molecular communication. Similarly, an analogous question is whether a particular nanoparticle responds in an appropriate way to a stimulus (chemical, light, magnetic, or temperature) or, after release of cargo, produces an appropriate response in a biological cell or in another nanoparticle.

On the other hand, a communications engineer is also concerned with the quality of communication between a sender and receiver. For example, an important goal is for a message to be reliably communicated from the sender to the receiver. Via the framework of Shannon, the quality of communication can be quantified via statistical information metrics. These quantitative metrics allow the optimization of the communication against various trade-offs regarding the available resources, the environmental or channel conditions, and the rate and accuracy of the information transfer. However, synthetic biologists and chemists have not been interested in understanding biological communication systems beyond whether or not they produce a desired response, and the Shannon framework has seen limited use up to this point. Moreover, communication engineers adapting theories from conventional electromagnetic communications to the biological communications often make too many simplifications for the sake of tractability and generalizability of their models and analyses, eventually degrading the physical correspondence of their work. This renders the developed models impractical to a large extent for the use of synthetic biologists who need to deal with the peculiarities of their biological designs in laboratory benches.

In a third direction, philosophers of science are interested in a rigorous conceptualization of biological communications. Such frameworks provide important clarifications into what should and should not be called communication as well as highlighting the limitations of various definitions of communication. However, the frameworks do not immediately lead to quantitative models of communication systems nor direct guidance for experimental design, but it can help scientists to acquire awareness about important conceptual ideas behind their work.

As a consequence of these different viewpoints, there are now significant gaps between experiments and mathematical/conceptual communication frameworks. This leads to difficulties in understanding how the tools and problems in each community can profitably interact. It is not immediately clear how the questions in biological communications relevant for synthetic biologists or chemists can be addressed by communication engineers or philosophers of science and vice versa.

## 5. Looking Forward

In this section, we highlight the main points of action to look forward when promoting interdisciplinary collaborations focused on molecular communications.

### 5.1. Developing a Common Language

Developing a common language appears to be the primary challenge in establishing strong interactions between experimental work in synthetic biology, nanotechnology and chemistry, and the mathematical/conceptual frameworks in communication engineering and philosophy of science. For a start, what does a biological communication, and specifically a chemical communication, system consist of?

It is also necessary to highlight that the synthetic biology community is large, including a variety of approaches. Similarly, within communications engineering, there are different specialities such as those focusing on ideas from information theory or signal processing. These differences within communities arise due to different problems or tools of interest. To have a strong interaction between communities, it is necessary for each of them to better understand which tools and problems within the others are likely to lead to progress.

To avoid disconnections between the communities, we believe it is important to give continuity to the discussion and debate with new events which foster collaborations among the scholars. The most efficient way to develop strong interactions is that both areas should have ways to grow together. We are also aware that developing truly inter-, cross-, and trans-disciplinary research is challenging. However, at the same time, there are examples in the history of science that show how fruitful these efforts can be. The intersection between molecular communications with nanoscience, nanotechnology and synthetic biology can be the arena for the next qualitative leap which can lead us to a next sci-tech paradigm (Internet of Bio-Nano Things, artificial life, smart drug delivery systems, etc.).

### 5.2. Developing Common Objectives

It is important to recognize that not all problems within a community will or should be of interest to another; therefore, it is crucial to identify problems where different communities can offer complementary tools and solutions.

In the fields of nanotechnology and synthetic biology, there are two basic objectives: designing systems with new capabilities or providing explanations for the behavior of existing systems that is not yet fully understood. Within communications engineering and the philosophy of science, there is inherent interest in developing new definitions and quantification of information. Interactions between these communities should therefore address these objectives.

For example, this could mean that the models underlying analysis within communication engineering should be more closely tied to and be interpretable by the synthetic biology, chemistry, and nanotechnology communities. On the other hand, to gain a better understanding of the nature of biological communications, it could be beneficial for synthetic biologists to provide communications engineers and philosophers of science with experimental systems, which can be used to develop mathematical and conceptual frameworks that are applicable to modern synthetic biological or chemical systems.

### 5.3. Supporting Emerging Synergies

More extensive interactions between the various communities interested in molecular communications are highly desirable. These interactions can be facilitated through tracks at conferences or special issues in journals inviting researchers from other communities as well as through the creation of new conferences and journals focused on interdisciplinary research at the intersection of these emerging fields. Additional cooperation initiatives (e.g., the European COST Actions) can also be useful in fostering collaboration. It is then of vital importance for the members of these communities to inform stakeholders about the potentialities of the convergence identified in this article. Funding agencies could then evaluate in a more informed manner and with a vision of the medium and long term the potential of this research arena in developing genuinely new technologies. The interest in the multidisciplinary research here described (that somehow resonates with the envisioned future shaped by Nano-Bio-Info-Cogno systems [58]) is expected to grow in next decades, and thus, it could be targeted for early attention by funding agencies. The reason is not only the potential progress it can deliver in science but also the strategic role it can play for technology and ultimately economy.

At present, synthetic biology devoted to the construction, from scratch, of cell-like systems and nanotechnology have been concerned with relatively simple systems. As these systems become better understood, it is expected that more complex ones will emerge. In this context, notions of information may play a more useful role. If the communities mentioned in this article begin interacting with each other while more complex systems emerge, there may be greater opportunities in the near future to have more successful interactions.

### 5.4. Training Interdisciplinary Researchers

Creating a new generation of researchers who have the interdisciplinary skills set necessary to tackle challenging research problems that span multiple fields can be instrumental in enabling the envisioned synergies. For example, researchers who have gained hands-on experience in bio/nanotechnology, coupled with a theoretical understanding of information and communication technologies (ICT), can effectively work on developing realistic experimental testbeds for molecular communication systems or practical artificial cell networks optimized from information and communication perspectives.

Young graduates and researchers who have not yet internalized the established practices and conventions of a particular discipline can have a high potential for integrating multiple perspectives into their research, especially when exposed to an interdisciplinary curriculum. Given their higher mobility, young researchers with interdisciplinary backgrounds can also be instrumental in transferring knowledge and experience among different groups and institutions, thereby facilitating interactions between different disciplines.

To this end, different research communities should come together to organize interdisciplinary summer schools, post-graduate courses, and similar programs, which could be possibly accompanied by the production of traditional or innovative teaching materials. The EU MSCA Doctoral Networks Program, which supports EU-wide consortiums of universities, research institutions, and companies in implementing new doctoral programs for the training of highly skilled doctoral candidates can be very useful in achieving these objectives.

## 6. Conclusions

In summary, we have presented a structured overview of the different perspectives and approaches of diverse fields on molecular communications and the potential synergies that can be achieved through their interactions. We have identified the challenges and the obstacles that presently hamper interdisciplinary collaboration in this field, and we proposed possible solutions to surmount them. By combining theoretical perspectives from communication engineering and philosophy of science with experimental approaches from synthetic biology and nanotechnology, we believe that new insights and opportunities in molecular communications can be unlocked. Going forward, we expect that future workshops and special issues will be beneficial in continuing the discussions and facilitate effective collaboration. 

## Data Availability

Not applicable.
